# Natural Compounds for the Treatment of Acute Pancreatitis: Novel Anti-Inflammatory Therapies

**DOI:** 10.3390/biom14091101

**Published:** 2024-09-02

**Authors:** Wenkai Jiang, Xiao Li, Yi Zhang, Wence Zhou

**Affiliations:** 1 The Second Clinical Medical College, Lanzhou University, Lanzhou 730030, China; jiangwk21@lzu.edu.cn (W.J.); lixiao21@lzu.edu.cn (X.L.); 2 The First Clinical Medical College, Lanzhou University, Lanzhou 730030, China; zhyi2021@lzu.edu.cn

**Keywords:** acute pancreatitis, anti-inflammation, MAPK, NF-κB, phytochemical

## Abstract

Acute pancreatitis remains a serious public health problem, and the burden of acute pancreatitis is increasing. With significant morbidity and serious complications, appropriate and effective therapies are critical. Great progress has been made in understanding the pathophysiology of acute pancreatitis over the past two decades. However, specific drugs targeting key molecules and pathways involved in acute pancreatitis still require further study. Natural compounds extracted from plants have a variety of biological activities and can inhibit inflammation and oxidative stress in acute pancreatitis by blocking several signaling pathways, such as the nuclear factor kappa-B and mitogen-activated protein kinase pathways. In this article, we review the therapeutic effects of various types of phytochemicals on acute pancreatitis and discuss the mechanism of action of these natural compounds in acute pancreatitis, aiming to provide clearer insights into the treatment of acute pancreatitis.

## 1. Introduction

Pancreatitis is a common digestive system disease characterized by pancreatic acinar cell necrosis and systemic inflammation [1]. In 2017, there were more than six million cases of pancreatitis worldwide [2]. When acute pancreatitis (AP) develops into severe acute pancreatitis (SAP), it seriously endangers patients’ lives and health [3]. The development of AP can be delayed by some therapies, such as fluid resuscitation, pain control, and nutritional support [4,5]. Owing to the limited efficacy of conventional treatment methods and the lack of effective targets for AP treatment, the current treatment for AP is a phased, multidisciplinary, and progressive symptomatic strategy.

Small-molecule compounds extracted from natural plants have a variety of biological activities [6,7]. These natural compounds can directly act on cancer cells, pathogens, or microorganisms while causing very little damage to normal cells [8,9,10]. Natural compounds have antioxidative effects and can inhibit the production of inflammatory mediators [11,12]. Thus, their application provides a new understanding of alleviating the inflammatory process and tissue damage of AP. In this article, we review the roles of various natural compounds in AP.

## 2. Characteristics of AP

### 2.1. Etiology and Clinical Features

The main causes of AP include pancreatic duct obstruction secondary to cholelithiasis, alcohol, endoscopic retrograde cholangiopancreatography, and pathological cell dysfunction triggered by various drugs [13]. Typical upper abdominal pain and abnormal increases in serum amylase or lipase may occur in the early stage of AP, and abnormal changes may also be observed in abdominal imaging [1]. When AP progresses to SAP, there are local or systemic complications [4,5]. Some patients develop new-onset diabetes or pancreatic exocrine insufficiency after AP [13]. In addition, approximately 17% of patients with AP experience recurrence, and 8% progress to chronic pancreatitis (CP) [14]. Owing to the atrophy of normal pancreatic tissue and replacement with fibrotic tissue, the endocrine and exocrine functions of the pancreas are progressively lost, leading to chronic abdominal pain, diabetes, and dyspepsia [15].

### 2.2. Pathophysiology and Molecular Mechanisms

**Figure 1:**
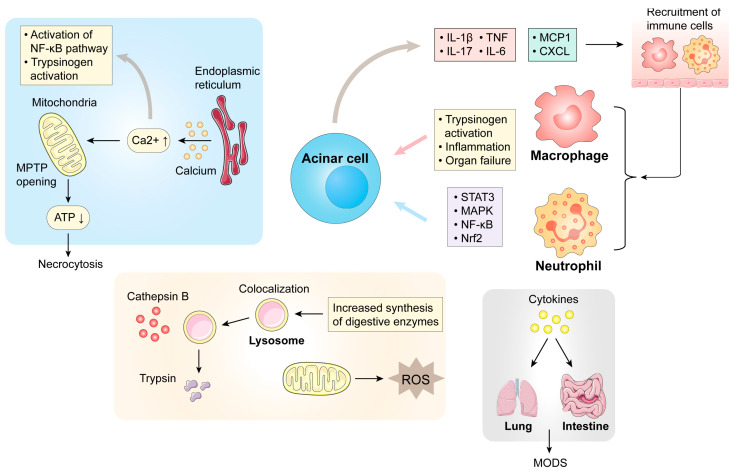
The mechanism of development of AP. The main mechanism of AP is the abnormal increase in the calcium concentration and abnormal activation of trypsinogen. In acinar cells, abnormal elevation of Ca^2+^ can lead to changes in the mitochondrial membrane potential, which leads to activation of the NF-κB pathway and trypsin, leading to necrosis of acinar cells. Lysosomes and zymogen granules colocalize, and cathepsin B activates trypsinogen by converting it into trypsin, which is released into the cytoplasm, leading to autodigestion. After acinar cell injury, some cytokines and chemokines can recruit inflammatory cells to the pancreatic tissue, further aggravating inflammation and injury to the pancreatic tissue through the signaling pathway, and even leading to the failure of other organs (lung and intestines), eventually leading to multiple organ dysfunction syndrome. The up arrow indicates elevated levels and the down arrow indicates decreased levels. Abbreviations: MPTP, mitochondrial permeability transition pore; MODS, multiple organ dysfunction syndrome; ROS, reactive oxygen species; NF-kB, nuclear factor kappa-B; ATP, adenosine triphosphate; IL, interleukin; TNF, tumor necrosis factor; MCP1, monocyte chemotactic Protein 1; CXCL, C-X-C ligand; STAT3, signal transducer and activator of transcription 3; MAPK, mitogen-activated protein kinase; Nrf2, nuclear factor E2-related factor 2.

Currently, the pathological mechanisms of AP include mitochondrial dysfunction, trypsin overactivation, and immune inflammation [16]. These pathological processes eventually damage pancreatic acinar cells and mediate a series of inflammatory processes. Organelle dysfunction in acinar cells plays an important role in AP, and dysfunction of one organelle may lead to disorders of others. An abnormal increase in calcium is central to AP pathogenesis [17]. Bile acids and alcohol can stimulate calcium release in acinar cells, increasing the concentration of intracellular calcium. An increase in calcium changes the mitochondrial membrane potential, leading to mitochondrial dysfunction [16,18]. The overactivation of trypsinogen is another important part of AP. Cathepsin B activates trypsinogen by converting it into trypsin, which is released into the cytoplasm, leading to autodigestion within and outside acinar cells (Figure 1) [19]. Endoplasmic reticulum (ER) stress refers to the accumulation of misfolded or unfolded proteins within the ER. Alcohol and its metabolites can reduce the cellular ability to process and recycle unwanted proteins and cause ER stress in acinar cells [13]. Additionally, impaired autophagy is also a characteristic of pancreatitis, caused by a decreased ability of lysosomes to degrade cargo [16].

The abnormal expression of specific molecules in AP may be potential therapeutic targets and target pathways. Once acinar cells are injured, various chemokines are released, and immune cells, including macrophages (which are recruited by monocyte chemoattractant protein 1) and neutrophils (which are recruited by CXCL 1), are recruited to the site of injury [13,20]. Monocyte chemoattractant protein 1 promotes the transport of inflammatory monocytes [13]. Activated monocytes activate the nuclear factor kappa-B (NF-κB) pathway, which further mediates the gene expression of inflammatory cytokines and chemokines, such as tumor necrosis factor (TNF), interleukin-1 (IL-1), IL-6, and IL-18 [13,21]. IL-6 has been shown to be correlated with the severity of AP [22]. The IL-6-mediated Janus kinase 2/signal transducer and activator of transcription 3 (JAK2/STAT3) signaling pathway and the nuclear factor E2-related factor 2 (Nrf2) oxidative stress response signaling pathway are activated [23]. Moreover, microRNAs (miRNAs) can regulate pancreatic acinar necrosis, apoptosis, and inflammation in AP [24]. Some miRNAs, such as miRNA-9, miRNA-155, and miRNA-148a, can regulate inflammation-related signaling pathways in the human body [25,26,27]. Additionally, some studies have shown that immunogenic cell death and autophagy-related genes are abnormally expressed in AP [28,29].

**Figure 2:**
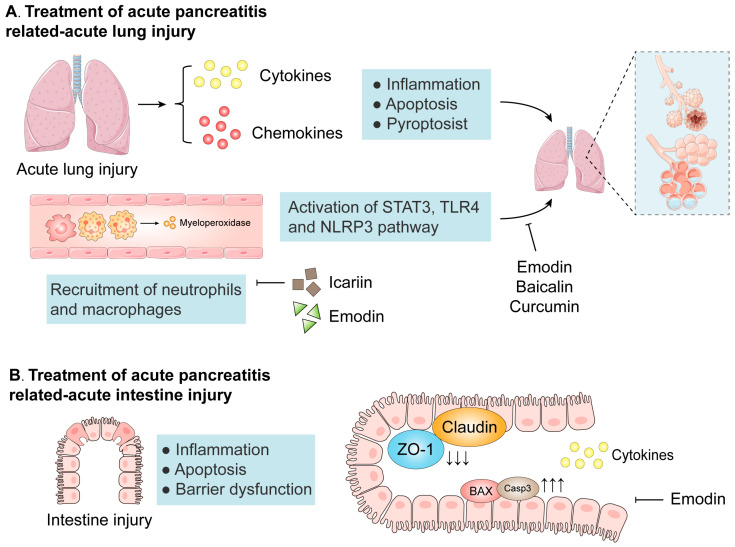
The mechanism of acute pancreatitis-related lung and intestine injury and their inhibition by natural compounds. Abbreviations:; STAT3, signal transducer and activator of transcription 3; ZO-1, zonula occludens-1; TLR4, Toll-like receptor 4; NLRP3, nod-like receptor protein 3; BAX, Bcl-2 associated X; Casp3, Caspase 3.

In addition to the local inflammation, systemic inflammation is more dangerous in AP (Figure 2). In the pulmonary microcirculation, a large number of inflammatory mediators and reactive oxygen species (ROS) destroy the blood–air barrier. Macrophages and neutrophils accumulate in the lungs, leading to a cascade of pathological changes in the pulmonary microcirculation [23]. The development of AP can be further worsened by pathological changes in the intestine, including intestinal barrier damage, ischemia–reperfusion injury, and changes in the microbiome. Intestinal mucosal injury is also a manifestation of systemic inflammation in AP [30]. In the early stage of SAP, increased colonization of *Escherichia coli* and *Streptococcus* leads to destruction of the mucus layer in the intestine [31]. As SAP progresses, the number of probiotics in the intestine decreases, and the chemical barrier is further damaged. The activation of inflammatory signaling pathways leads to the necrosis of epithelial cells, and tight junctions are damaged, causing abnormal immune responses in the intestine [31]. In addition, metabolites such as bile acids and lipopolysaccharides can aggravate AP, while short-chain fatty acids can alleviate AP through anti-inflammatory effects, intestinal epithelial barrier enhancement, and immunomodulatory effects [32]. The translocation of pathogenic bacteria caused by a damaged intestinal barrier can also cause inflammation in the lungs, resulting in lung injury [31]. Lung injury and intestinal injury jointly accelerate organ failure and ultimately result in multiple organ dysfunction syndrome.

## 3. Effects of Phytochemicals on AP

Despite recent progress in the treatment of AP, there are currently no specific drugs for treating AP or SAP. Many studies have focused on specific therapies for AP, including siRNAs targeting key molecules and inhibitors of inflammation-related pathways [33,34]. However, these methods are difficult to use in the clinic in the short term. Substances derived from plants have been used in the clinical treatment of AP in Asian countries [35,36]. The antioxidant effects of phytochemicals can inhibit inflammation-related pathways and the release of inflammatory mediators. This section elaborates on the inhibitory effects of various natural compounds (Table 1) on AP.

### 3.1. Terpenoids

Terpenoids are compounds composed of isoprene (C5) units and their derivatives, and they play important roles in plant growth and development [37,38]. Terpenoids include more than 40,000 structures and are used in the fragrance, chemical, and pharmaceutical industries [39]. According to the number of isoprene units, terpenoids can be divided into hemiterpeneoid (C5), monoterpenoids (C10), sesquiterpenoids (C15), diterpenoids (C20), triterpenoids (C30), and polyterpenes [40]. Some terpenoids, such as artemisinin (a bioactive compound of *Artemisia annua*) and paclitaxel (a diterpenoid compound isolated from *Taxus media*), have important medicinal properties [41,42].

Betulinic acid (BA) is a pentacyclic triterpenoid extracted from birch [43]. The effect of BA on pancreatitis mainly involves the inhibition of the NF-κB pathway. The NF-κB pathway is the classical inflammatory pathway. Normally, heterodimers composed of p50 and RelA are sequestered in the cytoplasm by IκB [44]. The core process of NF-κB activation is the phosphorylation of IκB. When the NF-κB pathway is activated, IκB is phosphorylated and degraded, and the released NF-κB dimers translocate to the nucleus and mediate the transcription of target genes [45]. In the AP model, BA treatment significantly inhibited the degradation of the IκBα protein and the translocation of NF-κB p65 to the nucleus, resulting in decreased levels of IL-1β, IL-6, TNF-α, and CCL2; in a mouse model, acinar cell necrosis and pancreatic tissue edema in mice are alleviated, and the activities of serum amylase and lipase are decreased in response to BA [46].

In pancreatic acinar cells, mixed-lineage kinase domain-like (MLKL) is phosphorylated by receptor-interacting protein kinase 3, leading to its oligomerization. MLKL oligomers translocate to the cell membrane, causing membrane rupture and cell necrosis [13]. Celastrol is another common pentacyclic triterpenoid extracted from the traditional Chinese medicine *Tripterygium wilfordii* Hook F. [47]. Celastrol significantly ameliorates the injury and necrosis of AP and decreases the levels of p-MLKL in the pancreas [48]. Another active ingredient extracted from *Tripterygium wilfordii* Hook F., triptolide, has also been shown to have anti-inflammatory and antioxidant effects on AP. Triptolide reduces the infiltration of neutrophils and macrophages in AP tissues, and triptolide pretreatment inhibits intracellular ROS levels in vivo [49].

Limonin is a tetracyclic triterpenoid with antibacterial, antiviral, and anti-inflammatory activities [50]. In mild AP and SAP mouse models, limonin mitigates the severity of AP and reduces the oxidative stress response in a dose-dependent manner, and these effects can be reversed by coumermycin A1 (a JAK activator), suggesting that limonin has protective effects on AP through suppressing the JAK2/STAT3 pathway [51]. 1,8-Cineole, a plant monoterpene with antioxidant properties, can also ameliorate AP. In mouse models, treatment with 1,8-cineole increases the level of IL-10 and reverses the increase in pancreatic myeloperoxidase (MPO) activity and malondialdehyde (MDA) levels caused by cerulein [52].

### 3.2. Alkaloids

Alkaloids are widely found in plants and are important natural products [53]. Alkaloids contain one or more basic nitrogen atoms, and more than 40,000 different alkaloid compounds have been identified [54]. The plants of *Berberidaceae*, *Liliaceae*, *Solanaceae*, and *Leguminosae* are rich in alkaloids [53]. Some alkaloids have been used in clinical practice. For example, ephedrine is used to treat respiratory diseases, and vinblastine and vincristine are used to treat cancer diseases [55,56,57].

Mitogen-activating proteins (MAPKs) are serine/threonine-protein kinases and regulate protein biosynthesis, the cell cycle, apoptosis, and differentiation [58]. Inhibiting the MAPK pathway is one of the current methods for treating inflammatory diseases [59]. Rutaecarpine significantly improved the viability of pancreatic acinar cells and inhibited apoptosis by increasing the expression of calcitonin gene-related peptides, thereby inhibiting the MAPK signaling pathway. Additionally, IL-10 is released to enhance the anti-inflammatory response after rutaecarpine treatment [60]. Piperine is an alkaloid present in black pepper that has many beneficial biological and pharmacological effects. Piperine has anti-inflammatory effects on AP and CP. In AP, piperine exerts anti-inflammatory effects mainly by inhibiting the activation of MAPKs [61]. Moreover, lower levels of the inflammatory factor IL-1β were observed in AP after treatment with piperine, and piperine also reduced ER stress, ER autophagy, and apoptosis in AP [62].

Berberine is an isoquinoline alkaloid that has been used in traditional Chinese medicine for many years [63]. Berberine has been shown to have a wide range of pharmacological effects. In AP, berberine significantly inhibited the activation of c-Jun N-terminal kinase (JNK) [64]. The JNK pathway is a MAPK signaling, and activated JNK can regulate apoptosis and inflammation [65]. Therefore, berberine-mediated JNK deactivation can improve AP and inhibit the expression of MPO and cytokines. Colchicine has the capacity to inhibit neutrophil adhesion and mobilization and has been approved for the treatment of gout [66]. In AP rat models, colchicine alleviates inflammatory responses by suppressing NF-κB, STAT3, and AKT signaling; additionally, colchicine reduces oxidative stress responses and apoptosis in pancreatic and lung tissues [67].

As a mediator of inflammation, TNF-α can regulate cell apoptosis and proliferation and promote the production of other chemokines and cytokines [68]. Interfering with the synergistic effect of TNF-α and IL-17A is an effective strategy for treating inflammatory diseases. In vitro, ellipticine inhibited the IL-17A- and TNF-α-induced activation of the NF-κB and MAPK signaling pathways, and the production of cytokines and chemokines was reduced in BEAS-2B lung epithelial cells. Ellipticine also reduces the activity of MPO in lung tissue, which is beneficial for reducing lung tissue inflammation caused by AP [69].

### 3.3. Flavonoids

Flavonoids are widely found in many vegetables and fruits, and researchers have identified more than 5000 flavonoid compounds [70,71,72]. Flavonoids have anti-inflammatory effects through different mechanisms, such as the inhibition of protein kinases and transcription factors, arachidonic acid metabolism, and immune cell activation [73]. Flavonoids are also an important class of natural antioxidants that have scavenger activity and can inhibit free radical production. Currently, flavonoids are used in the treatment of cancer, Alzheimer’s disease, and cardiovascular disease [74,75,76]. As mentioned previously, oxidative stress plays a role in the progression of AP. In various AP models, antioxidants can reduce inflammation in AP and delay the progression of AP caused by oxidative stress [77,78].

The Nrf2/heme oxygenase-1 (HO-1) pathway is a central regulator of the cellular antioxidant response [79]. Nrf2 is a nuclear antioxidant factor. In the case of oxidative stress, Nrf2 separates from Kelch-like ECH-associated protein 1 (Keap1) and can translocate to the nucleus. After entering the nucleus, Nrf2 interacts with HO-1 to inhibit the oxidative stress response [80]. In AP, the Nrf2 pathway is abnormally activated to exert antioxidant effects, but its function is limited. Glycyrrhizin, the active component of licorice, can inhibit ROS formation [81]. Isoliquiritigenin, a bioactive ingredient isolated from licorice, can decrease oxidative stress injury and significantly increase the expression of Nrf2/HO-1 [82]. Moreover, isoliquiritigenin plays a protective role in oxidative stress and inflammatory damage by regulating the Nrf2/NF-κB pathway in AP-related intestinal injury [83]. Dihydrokaempferol, a natural flavonoid extracted from *Bauhinia championii*, also affects the Nrf2 pathway in the AP model. Molecular docking showed that dihydrokaempferol can bind to the active site of the Keap1 protein; thus, dihydrokaempferol can be used as an agonist of the Keap1/Nrf2 pathway to reduce the oxidative stress response in AP [84]. The Nrf2/HO-1 pathway can also be regulated by pinocembrin, the main flavonoid in *propolis* [85].

Baicalin is a flavonoid compound in scutellarin [86]. In vivo, baicalin had a protective effect on AP in mice and significantly reduced the severity of pancreatic tissue injury; a further study showed that baicalin can inhibit necroptosis and p-MLKL oligomerization [87]. Toll-like receptor (TLR) 4 is a transmembrane receptor that plays an important role in complex intracellular inflammatory signaling pathways [88]. TLR4 activates NF-κB via MyD88, increasing pro-inflammatory cytokines [88]. Baicalin can decrease the levels of TLR4 and MyD88 in lung tissues by increasing the level of miRNA-182, resulting in alleviation of the inflammatory response and oxidative stress in acute lung injury (ALI) [89]. Another miRNA, miRNA-15a, is also a potential target for baicalein. Baicalein can regulate the levels of miRNA-15a and CDC42 to inhibit the JNK signaling pathway in AP [90]. Hypertriglyceridemia is one of the important causes of AP, and patients with severe hypertriglyceridemia are prone to developing SAP [91]. In a hypertriglyceridemia-induced AP model, baicalin regulated Nrf2/Keap1 signaling and decreased the activation of JAK2/STAT3 signaling to alleviate oxidative stress and inflammation [92].

Isorhamnetin is a naturally occurring flavonoid that can modulate the function of mitochondria [93]. Bioinformatics analysis revealed that isorhamnetin may be involved in the regulation of apoptosis and protein kinase activity. Isorhamnetin also inhibits mitochondrial injury and decreases ROS through its direct inhibition of the histone demethylation activity of KDM5B [94]. Another study showed that icariin can inhibit neutrophil infiltration into the pancreas and lungs and suppress the production of pro-inflammatory cytokines in the pancreas [95].

### 3.4. Other Phenolic Compounds

Resveratrol has anti-inflammatory, antiviral, antibacterial, and anti-tumor effects and is usually found in plants in *trans*-resveratrol form [96,97]. When administered orally, *trans*-resveratrol is converted to the more biologically active form of dihydro-resveratrol [98]. Resveratrol exerts therapeutic effects on interstitial lung disease, liver injury and fibrosis, cancer, and Alzheimer’s disease [99,100,101,102]. Resveratrol can regulate the actions of various cells in the pancreas. For example, resveratrol can inhibit ROS and miRNA-21-mediated glycolysis in pancreatic stellate cells [103]. In the AP model, resveratrol can stimulate the secretion of vascular endothelial growth factor A from bone marrow mesenchymal stem cells (BMSCs), activate the downstream phosphatidylinositol-4,5-diphosphate 3-kinase/protein kinase B signaling pathway, and inhibit pancreatic cell apoptosis [104].

In addition, resveratrol can reduce the microcirculation disorders caused by SAP. Resveratrol can inhibit the infiltration of immune cells and inflammatory cells into the vascular wall, which can reduce pathological smooth muscle cell proliferation, vascular inflammation, and vascular remodeling [105]. The SIRT1-FOXO axis has potential antioxidative stress activity in vascular endothelial cells [106]. Resveratrol can promote the interaction of SIRT1 and FOXO1, altering the function of vascular endothelial cells, while the expression of vascular endothelial growth factor and Ang II significantly decreases [107]. In vitro, resveratrol enhances the proliferation of human umbilical vein endothelial cells and promotes the regeneration of damaged blood vessels [104]. Although resveratrol has an inhibitory effect on AP progression, the oral absorption of resveratrol in humans is approximately 75%, and extensive metabolism in the gut and liver results in a bioavailability of less than 1% [108]. (R)-TML104 is a synthesized analog of resveratrol. Resveratrol plays an important role in inflammatory diseases by activating the AMPK-SIRT1 pathway [109]. Similarly, (R)-TML104 stimulates AMPK phosphorylation and upregulates SIRT1 expression in the pancreas. Activation of the IL-6-STAT3 pathway leads to high CCL2 expression in pancreatic tissues and neutrophil recruitment; however, STAT3 phosphorylation can be inhibited by (R)-TML104 [110].

Curcumin is a lipophilic compound extracted from turmeric and can reduce the severity of AP-induced inflammation through the MAPK signaling pathway [111]. Curcumin affects the p38 MAPK signaling pathway, through which it plays an anti-inflammatory role and regulates apoptosis [112]. AR42J cells are pancreatic acinar cells that can be stimulated into AP cells by a variety of substances in vitro [113]. Curcumin reduces the viability and amylase level of AR42J-induced AP cells and affects the phosphorylation of p38 in AP cells [111]. Acute kidney injury (AKI) is another serious complication of AP [114]. Curcumin can significantly reduce the levels of creatinine and blood urea nitrogen in SAP rats, downregulating the renal protein levels of JAK2 and STAT3 [115]. Moreover, researchers have developed curcumin-loaded poly (lactic-co-glycolic acid) microparticles, which can significantly reduce serum amylase and lipase levels. The effect of this drug delivery method is better than that of the oral or intraperitoneal injection of free drugs [116]. The slow release of natural products into the human body via microparticles is expected to lead to entry into clinical applications in the future.

### 3.5. Quinones

Emodin is found in many widely used Chinese herbs, such as *Rheum palmatum*, *Polygonum cuspidatum*, and *Polygonum multiflorum* [117]. The effect of emodin on AP is mainly to relieve lung injury. Alveolar macrophages are the main leukocytes in the respiratory tract and can secrete a variety of cytokines [118]. In ALI, pyroptosis in alveolar macrophages promotes the inflammatory response and alveolar cell injury; emodin can reduce the levels of inflammatory factors and lactate dehydrogenase to inhibit pyroptosis in alveolar macrophages, thereby reducing AP-related lung injury [119]. In rat alveolar macrophage NR8383 cells, emodin significantly reduces cold-inducible RNA-binding protein-activated NF-κB signaling and mitigates the IL-1β-dependent CXCL1 expression [120]. Nod-like receptor protein 3 (NLRP3) is one of the most studied inflammasomes [121]. The NLRP3 inflammasome leads to caspase 1-dependent release of the pro-inflammatory cytokine IL-1β, as well as pyroptotic cell death [122]. Emodin can significantly improve pulmonary edema and apoptosis in lung injury, and the expression levels of intercellular adhesion molecule-1 and NLRP3 are downregulated, thereby inhibiting the NLRP3-mediated recruitment of neutrophils to lung tissue [123]. Emodin can also inhibit AP-related lung injury through regulating the Nrf2/HO-1 signaling pathway [124].

Emodin can also alleviate AP-related intestinal injury. Network pharmacology analysis revealed that emodin is involved in the apoptosis signaling pathway and that the main targets of emodin are apoptotic proteins such as BAX, Bcl-2, and caspase 3; compared with the AP model, treatment with emodin can restore the expression of intestinal mucosal-barrier-related proteins and inhibit the expression of the proapoptotic proteins BAX and caspase 3, thereby protecting against intestinal mucosal barrier damage and inhibiting the apoptosis of intestinal epithelial cells [125]. Moreover, emodin can regulate the Notch1 pathway by regulating miRNA-218a-5p expression; in the intestinal tract, emodin increases the protein expression levels of occludin, zonula occludens-1, and E-cadherin to improve intestinal dysfunction [126].

**Figure 3:**
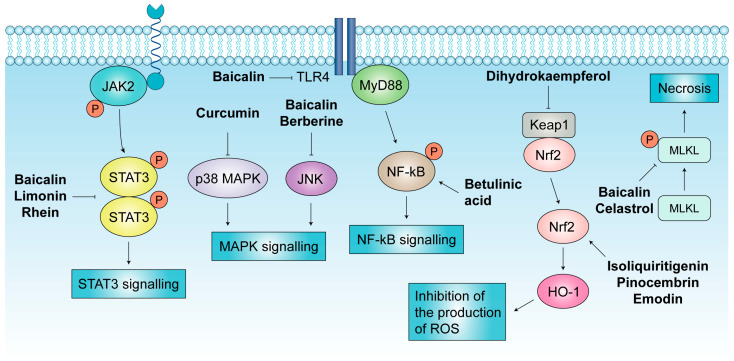
Activated intracellular signaling pathways in acute pancreatitis and their inhibition by natural compounds. Abbreviations: JAK2, Janus kinase 2; STAT3, signal transducer and activator of transcription 3; MAPK, mitogen-activating protein; TLR4, Toll-like receptor 4; JNK, c-Jun N-terminal kinase; NF-κB, nuclear factor kappa-B; Nrf2, nuclear factor E2-related factor 2; HO-1, heme oxygenase-1; ROS, reactive oxygen species; MLKL, mixed lineage kinase domain-like.

Rhein is one of the main active components of rhubarb and has anti-tumor, anti-fibrosis, antioxidant, and anti-inflammatory activity due to regulating various signaling pathways [127,128]. A study showed that rhubarb reversed mitochondrial damage and promoted pancreatic acinar proliferation; the treatment of 1 μM rhein to AR42J cells significantly decreased AMP-activated protein kinase expression and increased phosphoinositide 3-kinase and AKT expression [129]. The JAK/STAT3 pathway is one of the targets of rhein [127]. In AR42J cells and rat models, rhein inhibits the phosphorylation of JAK2 and STAT3, thereby reducing inflammation in AP [130]. The combination of rhein and other compounds also ameliorated pathological changes in the pancreas of AP mice [131]. The effects of natural compounds on various inflammation-related signaling pathways are summarized in Table 2 and Figure 3.

## 4. Areas of Future Development

### 4.1. Limitations and Challenges

The use of compounds derived from plants as drugs has certain limitations. Although plant compounds are inexpensive and nontoxic, the purification rate of phytochemical drugs is very low, which makes mass application difficult in clinical practice. Although chemical synthesis and microbial catalysis have been used in industrial production, the technological steps still need further improvement. For natural compounds with complex structures, the chemical synthesis method has the problems of a long synthesis route and low productivity, and expensive reagents are needed for separation, purification, and catalysis. Second, many natural compounds do not have high bioavailability, which limits their efficacy when used as drugs in the human body [132,133]. Third, there is little clinical evidence for the treatment of AP with natural compounds. Previous studies focused on the effects of herbal formulas, rather than single compounds on AP patients [134,135]. Although some natural compounds have been used in clinical trials (NCT01292005, NCT02947932, and NCT04989166), the practical use of natural compounds is still in the exploratory stage in clinical practice.

### 4.2. Improving in Future Directions

Future research should first focus on improving the bioavailability of natural compounds. The use of liposomes and nanoparticles loaded with drugs to improve biological activity is one of the current research directions [136]. Mannose-conjugated chitosan-coated lipid nano-capsules loaded with emodin have been used in SAP models and can allow emodin to selectively accumulate in the pancreas to promote the M2 phenotype polarization of macrophages [137]. A new drug delivery system using macrophage membrane-coated UiO-66-NH2 nanoparticles loaded with emodin also showed potential for prolonging drug circulation and ameliorating pancreatic tissue injury [138]. Compared with the use of the rhein alone, F127-modified liposomal rhein exhibited prolonged systemic circulation time and superior drug distribution in an AP rat model [139]. Cinnamic acid nanoparticles have antioxidant and anti-inflammatory effects and downregulate the NLRP3, NF-κB, and MAPK signaling pathways in AP rat models [140]. Moreover, fisetin-loaded lipid polymer hybrid nanoparticles have been shown to have protective effects on lung, liver, and renal injury and reduce the expression level of TLR4 in AP models [141]. Recently, a study investigating the effects of nano-curcumin supplementation on clinical outcomes in AP patients revealed that nano-curcumin is safe and may reduce the length of hospital stay (NCT04989166).

Second, the efficacy and safety of these natural compounds, including their ability to inhibit the inflammatory response and drug side effects, should be further evaluated. Although these natural compounds do little damage to normal cells in the short term, whether they have long-term side effects needs to be investigated.

Third, a variety of natural compounds have advantages in the treatment of other organ injuries in AP (Table 3). Because AP-related ALI and AKI are fatal, it is necessary to further investigate the mechanism by which natural compounds alleviate AP complications in the lungs, kidneys, and other organs.

## 5. Summary

Bioactive compounds extracted from plants can inhibit related cellular pathways through a variety of anti-inflammatory mechanisms, thereby antagonizing oxidative stress and inhibiting the release of inflammatory factors to treat AP. Exploring the mechanism and specific targets of these compounds is the main focus of current research. The application of molecular docking technology and network pharmacology will provide specific targets and functional pathways for these natural compounds. In conclusion, natural compounds have broad research potential and application prospects in the treatment of AP.

## Figures and Tables

**Table 1:** Natural compounds for treating acute pancreatitis.

Compounds	Main Sources	Chemical Structures
Betulinic acid	*Birch*	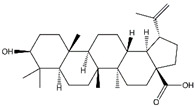
Celastrol	*Tripterygium wilfordii*	* 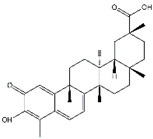 *
Triptolide	*Tripterygium wilfordii*	* 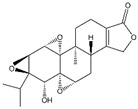 *
Limonin	*Citrus fruits*	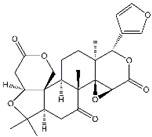
Rutaecarpine	*Evodia rutaecarpa*	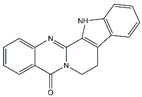
Piperine	*Black pepper*	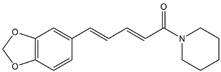
Berberine	*Coptis chinensis*	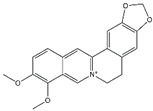
Colchicine	*Autumn crocus*	* 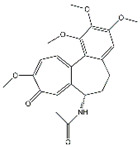 *
Ellipticine	*Ochrosia elliptica*	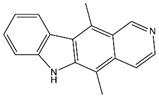
Isoliquiritigenin	*Licorice*	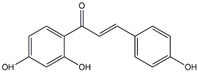
Dihydrokaempferol	*Bauhinia championii*	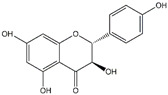
Pinocembrin	*Propolis*	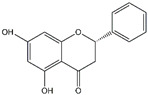
Baicalin	*Scutellaria baicalensis*	* 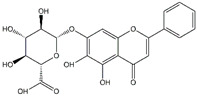 *
Baicalein	*Scutellaria baicalensis*	* 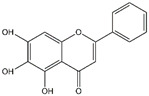 *
Icariin	*Epimedium*	** 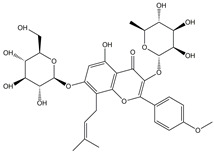 **
Isorhamnetin	*Hippophae rhamnoides* L.	* 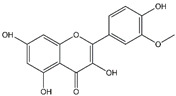 *
Resveratrol	*Grapes*	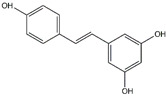
Curcumin	*Turmeric*	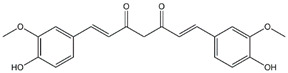
Emodin	*Rheum palmatum*	* 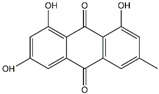 *
Rhein	*Rheum palmatum*	* 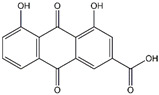 *

**Table 2:** Summary of natural compounds in various inflammation-related signaling pathways.

Signaling Pathways	Compounds	Effects	Ref.
MAPK	Rutaecarpine	Reduction of IL-6 and TNF-α, and increasing in IL-10	[60]
	Berberine	Inhibition of JNK activation and reduction of iNOS	[64]
	Ellipticine	Reduction of IL-17A	[69]
	Curcumin	Reduction of IL-6, TNF-α and C-reactive protein	[111]
NF-κB	Betulinic acid	Inhibition of the infiltration of neutrophils and macrophages	[46]
	Colchicine	Reduction of iNOS and ROS production	[67]
	Ellipticine	Reduction of IL-17A	[69]
	Isoliquiritigenin	Inhibition of oxidative stress	[83]
JAK/STAT3	Limonin	Reduction of IL-6 and TNF-α	[51]
	Colchicine	Reduction of iNOS and ROS production	[67]
	Baicalin	Inhibition of apoptosis, inflammation and oxidative stress	[92]
	Rhein	Reduction of IL-6 and TNF-α	[130]
Nrf2/HO-1	Triptolide	Inhibition of the infiltration of neutrophils and macrophages	[49]
	Isoliquiritigenin	Inhibition of oxidative stress and inflammation	[82,83]
	Dihydrokaempferol	Inhibition of oxidative stress	[84]
	Emodin	Reduction of IL-6 and TNF-α	[124]

Abbreviations: Ref, reference; IL, interleukin; JNK, c-Jun N-terminal kinase; iNOS, inducible nitric oxide synthase; ROS, reactive oxygen species; TNF, tumor necrosis factor.

**Table 3:** Summary of natural compounds used for the treatment of acute-pancreatitis-related complications in lungs, small intestine, and kidneys.

Compounds	Effects	Model	Ref.
Colchicine	Suppressing NF-κB, STAT3 and AKT signaling pathways in lung tissue	Rat model	[67]
Ellipticine	Inhibition of IL-17A- and TNF-α	BEAS-2B cell	[69]
Baicalin	Inhibition of TLR4/MyD88 signaling pathway	Rat model	[89]
Icariin	Inhibited neutrophil infiltration of lung	Mouse model	[95]
Curcumin	Reduction of creatinine and blood urea nitrogen and suppression of the JAK2/STAT3 pathway in kidney tissues	Rat model	[115]
Emodin	Inhibition of pyroptosis in alveolar macrophages	Rat and mouse model	[119]
	Inhibition of NLRP3-mediated recruitment of neutrophils to lung	Rat model	[123]
	Inhibition of Nrf2/HO-1 signaling pathway	Rat alveolar epithelial cell line L2 cells	[124]
	Increasing of the expression of intestinal barrier-related proteins and regulation of T helper cells	Mouse model	[125]
	Regulating Notch1 and pathways by regulating the miRNA-218a-5p expression in the intestine	Rat model and IEC-6 cell	[126]
Isoliquiritigenin	Regulating the Nrf2/NF-κB pathway in the intestine	Mouse model	[83]

Abbreviations: Ref, reference; NF-κB, nuclear factor kappa-B; STAT3, signal transducer and activator of transcription 3; IL, interleukin; TNF, tumor necrosis factor; TLR4, Toll-like receptor 4; miRNA: microRNA; JAK, Janus kinase 2; NLRP3, Nod-like receptor protein 3; Nrf2, nuclear factor E2-related factor 2; HO-1, heme oxygenase-1.
